# Vasodilatory Effects and Mechanisms of Action of *Bacopa monnieri* Active Compounds on Rat Mesenteric Arteries

**DOI:** 10.3390/molecules24122243

**Published:** 2019-06-15

**Authors:** Natakorn Kamkaew, Tamkeen Urooj Paracha, Kornkanok Ingkaninan, Neti Waranuch, Krongkarn Chootip

**Affiliations:** 1 Department of Physiology, Faculty of Medical Science, Naresuan University, Phitsanulok 65000, Thailand; natakornk@gmail.com; 2 Division of Physiology, School of Medical Sciences, University of Phayao, Phayao 56000, Thailand; 3 Department of Pharmacy Practice, Faculty of Pharmaceutical Sciences, Naresuan University, Phitsanulok 65000, Thailand; ctamz@hotmail.com; 4 Department of Pharmaceutical Chemistry and Pharmacognosy, Faculty of Pharmaceutical Sciences and Center of Excellence for Innovation in Chemistry, Naresuan University, Phitsanulok 65000, Thailand; kornkanoki@nu.ac.th; 5 Cosmetics and Natural Products Research Center, Department of Pharmaceutical Technology and Center of Excellence for Innovation in Chemistry, Faculty of Pharmaceutical Sciences, Naresuan University, Phitsanulok 65000, Thailand; netiw@nu.ac.th

**Keywords:** luteolin, apigenin, bacoside A, bacopaside I, vasorelaxation

## Abstract

*B. monnieri* extract (BME) is an abundant source of bioactive compounds, including saponins and flavonoids known to produce vasodilation. However, it is unclear which components are the more effective vasodilators. The aim of this research was to investigate the vasorelaxant effects and mechanisms of action of saponins and flavonoids on rat isolated mesenteric arteries using the organ bath technique. The vasorelaxant mechanisms, including endothelial nitric oxide synthase (eNOS) pathway and calcium flux were examined. Saponins (bacoside A and bacopaside I), and flavonoids (luteolin and apigenin) at 0.1–100 µM caused vasorelaxation in a concentration-dependent manner. Luteolin and apigenin produced vasorelaxation in endothelial intact vessels with more efficacy (E_max_ 99.4 ± 0.7 and 95.3 ± 2.6%) and potency (EC_50_ 4.35 ± 1.31 and 8.93 ± 3.33 µM) than bacoside A and bacopaside I (E_max_ 83.6 ± 2.9 and 79.9 ± 8.2%; EC_50_ 10.8 ± 5.9 and 14.6 ± 5.4 µM). Pretreatment of endothelial intact rings, with L-NAME (100 µM); an eNOS inhibitor, or removal of the endothelium reduced the relaxant effects of all compounds. In K^+^-depolarised vessels suspended in Ca^2+^-free solution, these active compounds inhibited CaCl_2_-induced contraction in endothelial denuded arterial rings. Moreover, the active compounds attenuated transient contractions induced by 10 µM phenylephrine in Ca^2+^-free medium containing EGTA (1 mM). Thus, relaxant effects occurred in both endothelial intact and denuded vessels which signify actions through both endothelium and vascular smooth muscle cells. In conclusion, the flavonoids have about twice the potency of saponins as vasodilators. However, in the BME, there is ~20 × the amount of vaso-reactive saponins and thus are more effective.

## 1. Introduction

*Bacopa monnieri* (L.) Wettst. or Brahmi, is an Ayurvedic medicine traditionally used as a memory enhancer. Along with memory improvement, it is known to promote mental health, as a neurotonic and cardiotonic agent. *B. monnieri* extract (BME) clearly has a cognitive enhancing potential and neuroprotective effects [1,2,3,4,5,6,7,8,9,10,11,12,13,14,15,16]. It has been shown to be antioxidant in rat brain [17,18] and to possess several pharmacological actions such as anti-depressant [19,20,21], anti-dementia [9], anti-cholinesterase [8,9], anti-hyperglycaemic [22] and anti-hyperlipidaemia [23]. *B. monnieri* appears to be non-toxic using haematological and blood biochemical diagnostics [24,25,26]. BME demonstrated cardioprotection, improved coronary blood flow, and protection against myocardial ischemia reperfusion injury [27,28]. Our recent work showed that BME acted as a vasodilator by releasing nitric oxide (NO) from endothelium and inhibiting Ca^2+^ influx and Ca^2+^ release from the sarcoplasmic reticulum (SR). These mechanisms mediated an acute decrease in blood pressure [29]. Also, daily oral BME (40 mg/kg) in rats for 8 weeks showed a significant increase in cerebral blood flow [30], which implies cerebrovascular dilation.

BME contains an abundance of bioactive compounds. They include dammarane-type triterpenoid saponins, jujubogenin and pseudojujubogenin glycosides. These saponins are predominantly bacopaside I and bacoside A, a mixture of bacoside A_3_, bacopaside II, jujubogenin isomer of bacopasaponin C, and bacopasaponin C [31,32,33]. Other than saponins, flavonoids, essentially luteolin and apigenin are also present in *B. monnieri* [10,34,35,36]. Bacoside A_3_ and bacopaside II relax rat mesenteric arteries [29] but the mechanism(s) of their relaxation are presently unknown. The flavonoids found in *B. monnieri* also relax rat aortae [37,38,39,40,41] but these experiments used a variety of protocols and vascular preparations. Therefore, it is important to make a side-by-side comparison of these flavonoids with the *B. monnieri* saponins using a resistance vessel type. For this we choose the mesenteric artery which better exemplifies actions on regional blood flow and systemic blood pressure than the aorta. This work provides evidence to clarify the effective *B. monnieri* components for vasorelaxation which could be related to the improvement of blood flow or memory enhancement.

## 2. Results

### 2.1. Vasorelaxant Effects of the B. monnieri Active Compounds

**Figure 1:**
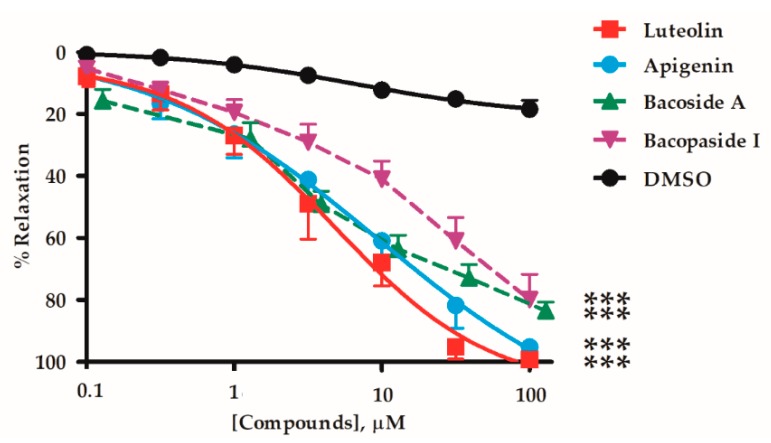
Relaxations induced by luteolin, apigenin, bacoside A, and bacopaside I (0.1–100 µM) and vehicle (DMSO) in endothelial intact mesenteric arteries precontracted with phenylephrine (10 µM). Values are mean ± SEM of 6–9 individual arterial rings. *** indicates *p* < 0.001 comparing relaxation for each compound with the control (DMSO) using two-way ANOVA (n = 6–9). Lines were fitted by non-linear regression.

Mesenteric arteries of rats were isolated and mounted in an organ bath via intraluminal wire hooks connected to a force transducer. The vessels were pre-contracted with 10 µM phenylephrine (PE), before adding *B. monnieri* compounds including flavonoids (luteolin and apigenin), bacopaside I, and the saponin mixture (bacoside A) at 0.1–100 µM. *B. monnieri* compounds caused vasorelaxation of endothelial intact arteries (+EC) in a concentration-dependent manner (Figure 1) with EC_50_ and E_max_ values shown in Table 1.

### 2.2. Mechanisms of Vasorelaxation by B. monnieri Compounds

**Figure 2:**
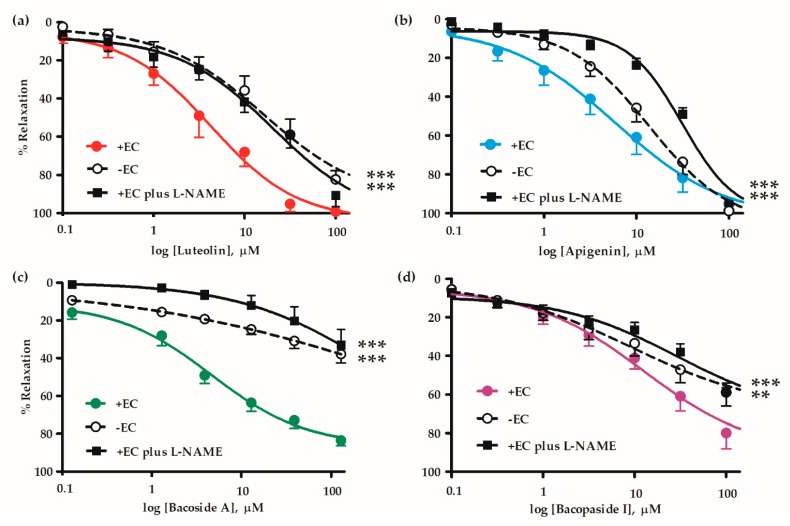
Cumulative concentration-response curves of (**a**) luteolin, (**b**) apigenin, (**c**) bacoside A and (**d**) bacopaside I in concentrations (0.1–100 µM) in endothelial intact (+EC), denuded (-EC) mesenteric arterial rings and endothelial intact vessels pre-incubated in L-NAME (100 µM). The graphs are expressed as %relaxation of vessel pre-contracted with 10 µM PE. Values are mean ± SEM of 6–9 individual arteries. ** *p* < 0.01, *** *p* < 0.001 each compound compared with intact vessels (+EC) using two-way ANOVA (n = 6–9).

All the *B. monnieri* compounds caused vasorelaxation in both endothelial intact (+EC) and endothelial denuded (-EC) mesenteric arterial rings. The relaxations were reduced by the removal of endothelium, implying that these compounds acted via an effect on endothelial vasodilators. However, the compounds still produced some vasorelaxations of the endothelial denuded arterial rings due to a direct action on vascular smooth muscle cells. For intact vessels, L-NAME (inhibitor of endothelial NO synthase; eNOS inhibitor), also reduced the vasorelaxations (Figure 2, Table 2). These reductions suggest that some or all the vasorelaxations were mediated through production and release of NO by endothelial cells.

### 2.3. B. monnieri Compounds and Ca^2+^ Influx

**Figure 3:**
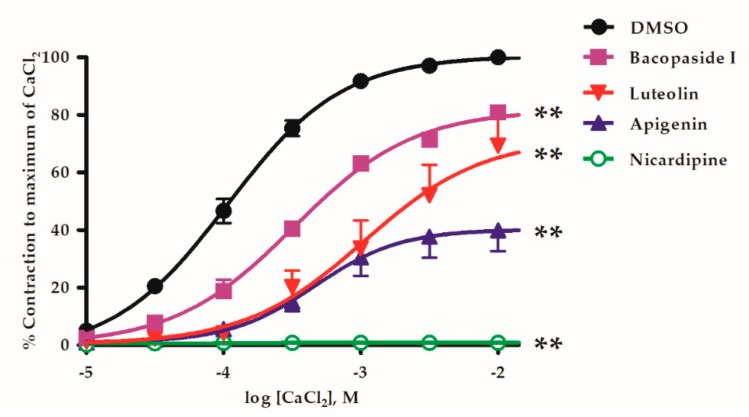
CaCl_2_-induced contractions of denuded mesenteric arteries pre-incubated in high K^+^, Ca^2+^-free media in the conditions of pre-incubation with DMSO (negative control), 10 µM bacopaside I, 10 µM luteolin, 10 µM apigenin, and 1 µM nicardipine (positive control). *Y*-axis, % contraction compared to the contraction achieved with the highest Ca^2+^ concentration during the initial run without a *B. monnieri* compound in the same vessel. Values are mean ± SEM of 4–6 individual arteries. ** *p* < 0.01 each of the active compounds compared to DMSO using two-way ANOVA (n = 4–6).

Voltage-operated Ca^2+^ channels (VOCCs) were activated by depolarising denuded vessels with 80 mM K^+^ in Ca^2+^-free Krebs’ solution. Then vascular contraction elicited by CaCl_2_ accumulatively added at increasing concentrations (0.01–10 mM). In the same vessel, the protocol was repeated by pre-incubation with 10 µM *B. monnieri* compounds for 15 min and these CaCl_2_-induced contractions were inhibited and seen as a rightward shift of the plots and reduced E_max_ from control (Figure 3).

The maximum contraction (E_max_) of control, bacopaside I, luteolin and apigenin were 100 ± 1.3, 81.9 ± 1.7, 72.0 ± 6.7 and 40.2 ± 3.5%, respectively. Positive control, L-type Ca^2+^-channel blocker, nicardipine (1 µM) completely abolished this CaCl_2_-induced vasoconstriction (Figure 3).

### 2.4. B. monnieri Compounds and Intracellular Ca^2+^ Release

**Figure 4:**
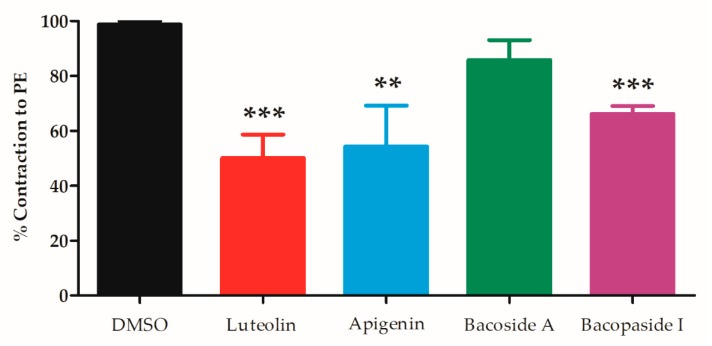
PE-induced contraction induced by Ca^2+^ release from sarcoplasmic reticulum of endothelial denuded mesenteric arteries in the presence of DMSO (control), 10 µM of luteolin, apigenin, bacoside A and bacopaside I. The data is % contraction to 10 µM PE induced contraction compared to contractions produced by the initial protocol without test compound. Values are mean ± SEM of 5–6 individual arteries. ** *p* < 0.01, *** *p* < 0.001 each of the active compounds compared with control using unpaired Student’s *t*-test (n = 5–6).

The release of intracellular Ca^2+^ from the sarcoplasmic reticulum is another important trigger of vascular contraction. Denuded arterial rings were pre-incubated in Ca^2+^-free Krebs’ solution for 10 min and then 10 µM PE added thereby eliciting a transient contraction. Then the protocol was repeated with the same arterial ring in the presence of the test compounds (control, apigenin, luteolin, bacoside A and bacopaside I) producing reduced contractions (98.8 ± 1.2, 50.1 ± 8.5, 54.3 ± 14.9, 85.8 ± 7.2 and 66.2 ± 2.9%, respectively) (Figure 4). Luteolin, apigenin and bacopaside I caused significant decrease in PE-induced contraction compared to the vehicle control (*p* < 0.001, <0.01 and <0.001, respectively).

## 3. Discussion

This is the first study comparing the vasodilatory mechanisms elicited by saponins (particularly bacoside A and bacopaside I) and the principal flavonoids (luteolin and apigenin) were the most potent (EC_50_ 4.4 and 8.9 µM) (Figure 1). However, these are present in BME at only about 1/20th the contents of the bacoside A saponins and bacopaside I (Figure S1 and Table S1) [42]. Thus in terms of the overall actions of the complete BME, the saponins would be expected to make a larger contribution to the vasorelaxation than the flavonoids.

However, higher potency of aglycone flavonoids compared to saponin glycosides may be due to sugar moieties interfering with the molecule interacting with the binding sites responsible for the vasorelaxation as suggested by previously, i.e., lipophilic groups in the ring skeleton of flavonoids increased their vasorelaxant activity [43]. This provides a basis for study of the molecular mechanisms of vasorelaxation of flavonoids.

We investigated the mechanisms of flavonoid- and saponin-induced relaxation by endothelial denudation in mesenteric arterial rings which impaired vasorelaxation (Figure 2). Role of NO was investigated using the eNOS inhibitor (L-NAME) with the test compounds. L-NAME increased EC_50_ and reduced E_max_ which imitated the effect of endothelial denudation, suggesting the relaxation was mainly medicated by NO. This accords with observations made by Jin et al. that a cyclooxygenase (COX) inhibitor did not affect the relaxation induced by apigenin [44], and consistent with our previous study of *B. monnieri* extract, where indomethacin had no effect on vasorelaxation [29]. There were some important concentration dependent differences between flavonoids and saponins. Firstly, denudation or blockade of eNOS reduced the effect of bacoside A more than bacopaside I, luteolin and apigenin. Perhaps this was a reflection of bacoside A being a mixture of saponins. However, curiously the responses of luteolin and apigenin to denudation and L-NAME where the latter had a greater effect.

Vascular smooth muscle express plasma membrane L-type Ca^2+^ channels that allow depolarisation dependent Ca^2+^ entry to trigger contraction. All three compounds (luteolin, apigenin and bacopaside I) tested in denuded vessels depressed this mechanism of contraction that can also explain in part, the vasorelaxant effect. But here, apigenin appeared to be more effective than luteolin while it was less effective in relaxation studies suggesting some heterogeneity in the mechanism of flavonoid action.

Ca^2+^ release from intracellular stores also regulates contraction via inositol trisphosphate (IP_3_) or ryanodine receptors (RyR) associated channels in the SR membranes. IP_3_ associated channels are commonly activated by plasma membrane G-protein coupled receptors including α_1_-receptors which are activated by PE. RyR channels are activated by Ca^2+^ itself. The three pure compounds also inhibited Ca^2+^ released from stores which can account for at least some vasorelaxation of vessels precontracted by PE. However, the bacoside A was without clear effect again suggesting some heterogeneity between the four test substances. Other Ca^2+^-channels may also be involved, for example T-channels and TRP channels, especially TRPC4 which is activated by α_1_-receptor activation.

K^+^ channels also play a role in regulation of vascular tone, i.e., voltage-dependent K^+^ (K_v_) channels open upon depolarization of the plasma membrane in vascular smooth muscle cells, and thus inhibits Ca^2+^ influx through VOCCs, resulting in vasodilation [45]. Jiang et al. also reported that luteolin inhibited Ca^2+^ channels, inhibited release of stored Ca^2+^ while K^+^ channels were activated, specifically via K_ATP_, K_Ca_, K_V_ and K_IR_ [40] therefore the effects of apigenin, bacoside A and bacopaside I involving K^+^ channels deserve further investigation. Our findings support those of Si et al. that luteolin can directly act on vascular endothelial cells, by inducing eNOS phosphorylation at Ser1177, leading to NO production [41]. The flavonoids evoke relaxations and also protect endothelial dependent vasorelaxation against oxidative stress [44,46,47] and diabetes [48], however vasoprotective effects of saponins needs further comprehensive investigation.

## 4. Materials and Methods

### 4.1. General Information

Tissues were from male Wistar rats (200–300 g) which were obtained from Nomura Siam International Co. Ltd. (Bangkok, Thailand). Experiments were approved by the Naresuan University Animal Care and Use Committee (NUACUC), protocol number NU-AE 600710. The rats were housed under the environmental conditions at 22 ± 1 °C, 12-h light and dark cycle, fed with standard rodent diet and tap water in Naresuan University Center for Animal Research (NUCAR) according to the guidelines for care and use of laboratory animals (Institute of Laboratory Animal Research, eighth edition 2011. Rats were anesthetized by intraperitoneal injection of thiopental sodium (100 mg/kg BW) and killed. The mesenteric arteries were excised, cleaned of surrounding loose connective tissue and cut into rings of 3–5 mm width. In some experiments, endothelial cells were mechanically removed by gently rubbing the lumen with a stainless steel wire. The mesenteric rings were mounted on a pair of intraluminal wires in organ chambers containing physiological Krebs’ solution (mM): NaCl, 122; KCl, 5; [N-(2-hydroxyethyl) piperazine N’-(2-ethanesulfonic acid)] HEPES, 10; KH_2_PO_4_, 0.5; NaH_2_PO_4_, 0.5; MgCl_2_, 1; glucose, 11; and CaCl_2_, 1.8 (pH 7.3), at 37 °C and aerated [29,49,50,51]. The vessel segments were allowed to equilibrate for 1-h at a resting tension of 1–1.3 g during which the solution was replaced every 15 min. Changes in isometric tension were measured using force transducer lever (CB Sciences Inc., Milford, MA, USA) connected to a MacLab A/D converter (Chart V7; A.D. Instruments, Castle Hill, NSW, Australia), stored and displayed on a personal computer. Following stabilization, the arterial rings were tested for viability by the application of 10 µM PE. Upon development of a steady contraction, the endothelium status was tested with 10 µM ACh. The vessel was considered endothelial intact when the ACh induced >70% relaxation. After establishing the status of the endothelium, the rings were then rinsed with Krebs’ solution for 30 min and one of the following protocols was initiated. Luteolin (lot 126M4061V) and apigenin (lot WE445301/1) were purchased from Sigma Aldrich (St. Louis, MO, USA). Bacoside A (lot 00002005-003) and bacopaside I (lot 00002002-T17H) were purchased from ChromaDex, Inc. (Irvine, CA, USA).

### 4.2. Vasorelaxant Effects of B. monnieri Active Compounds on Endothelial Intact Arteries

Following stabilization, endothelial intact rings of mesenteric arteries were pre-contracted with 10 µM PE. After the contraction had become constant, the *B. monnieri* active compounds (0.1–100 µM), including luteolin, apigenin, bacoside A or bacopaside I were added cumulatively.

### 4.3. Vasorelaxant Effects of B. monnieri Active Compounds on Endothelial Denuded Arteries

Successful endothelial denudation was confirmed by the absence of relaxation upon addition of 10 µM ACh. For investigation of the role of endothelium in 0.1–100 µM *B. monnieri* active compounds (luteolin, apigenin, bacoside A or bacopaside I) induced vasorelaxation, endothelial denuded arteries were used. The data of effect of active compounds were presented as %relaxation.

### 4.4. Study of Vasorelaxant Mechanisms of B. monnieri Active Compounds via eNOS Pathway

The role of the endothelial relaxing factor, NO, in *B. monnieri* active compounds (luteolin, apigenin, bacoside A or bacopaside I) induced vasorelaxation were evaluated in endothelial intact ring pre-treated with N^G^-nitro-L-arginine methyl ester (L-NAME, 100 µM), an inhibitor of eNOS, for 30 min prior to 10 µM PE exposure.

### 4.5. Study of Vasorelaxant Mechanisms of B. monnieri Active Compounds on Extracellular Ca^2+^ Influx

Endothelial denuded mesenteric arteries were equilibrated in Ca^2+^-free Krebs’ solution (containing (mM): ethylene glycol-bis (ß-aminoethyl ether)-N,N,N,N tetra acetic acid (EGTA), 0.01; NaCl, 122; KCl, 5; HEPES, 10; KH_2_PO_4_, 0.5; NaH_2_PO_4_, 0.5; MgCl_2_, 1 and glucose, 11 (pH 7.3)) for 30 min followed by replacing with Ca^2+^-free Krebs’ solution containing 80 mM K^+^ for 10 min which depolarizes the vascular smooth muscle cells, thus opening VOCCs. Various concentrations of CaCl_2_ were then added (0.01–10 mM) in a logarithmic progression. After obtaining the maximum response, the baths were washed out and replenished with Ca^2+^-free Krebs’ solution for 30 min. The Ca^2+^-free 80 mM K^+^ solution was then re-applied following pre-incubation for 10 min with either: 10 µM active compounds or 1 µM nicardipine (antagonist of VOCCs). Concentration-response curves to cumulative addition of CaCl_2_ were then repeated and compared with maximum contraction evoked by previous control CaCl_2_ challenges.

### 4.6. Study of Vasorelaxant Mechanisms of B. monnieri Active Compounds on Intracellular Ca^2+^ Release

To stimulate initial Ca^2+^ loading of the SR Ca^2+^ stores, endothelial denuded mesenteric arteries were exposed to 80 mM K^+^ solution for 5 min, and then washed out with Ca^2+^-free Krebs’ solution containing 1 mM EGTA for 10 min. The arterial rings were then challenged with 10 µM PE (acting through phospholipase C/IP_3_ signaling) which release Ca^2+^ from the SR thereby eliciting a transient contraction [29]. The same protocol was then repeated to ensure that similar transient contractions to PE could be obtained. Then, the arterial rings were challenged again with 80 mM K^+^ solution for 5 min, and washed out with Ca^2+^-free Krebs’ solution containing 1 mM EGTA and 10 µM active compounds for 10 min. The arterial rings were again challenged with 10 µM PE. The PE-induced contractions were compared in the presence or absence of active compounds.

### 4.7. Statistical Analyses

Statistical analyses used GraphPad Prism version 5.00 for Windows, (GraphPad Software Inc., La Jolla, CA, USA). Data from each concentration-effect curve was analysed using non-repeated two-way ANOVA. Curve fitting in the figures was generated by the same software using non-linear regression. EC_50_ and E_max_ were compared using unpaired Student’s *t* test. Values are expressed as mean ± SEM. A *p*-value < 0.05 was considered significant. ‘n’ is the number of vascular rings used, each ring originating from a different animal.

## 5. Conclusions

This study demonstrated that *B. monnieri* active components, including both saponins and flavonoids, produced vasodilatory effects on rat isolated mesenteric arteries partially via endothelial dependent release of vasodilators and also by direct effects on vascular smooth muscle cells via blockade of Ca^2+^ influx and its release from SR. This study for the first time reports the comparative vasodilatory effects of saponins and flavonoids found in *B. monnieri* extract. However, *B. monnieri* extract, flavonoids i.e., luteolin and apigenin would be more potent vasodilators but saponins have a greater effect because of their greater contents. Accordingly, the clinical benefits on enhanced blood flow and cognitive function may arise from a combination of flavonoids and particularly the saponins.

## Figures and Tables

**Table 1:** The EC_50_ and E_max_ of *B. monnieri* active compounds on relaxation of endothelial intact rat mesenteric arteries.

Active Compounds		EC_50_ (µM)	E_max_ (%)	n	*p*-Value Whole Graph Curves
**Flavonoids**	Luteolin	4.35 ± 1.31	99.4 ± 0.7	6	-
	Apigenin	8.93 ± 3.33	95.3 ± 2.6	9	NS
**Saponins**	Bacoside A	10.8 ± 5.9	83.6 ± 2.9 ††	7	< 0.05 †
	Bacopaside I	14.6 ± 5.4	79.9 ± 8.2 †	7	< 0.01 ††
**Vehicle**	DMSO	-	17.4 ± 3.1 ††	7	< 0.01 ††

Significantly different compared with luteolin † *p* < 0.05, †† *p* < 0.01 using unpaired Student’s *t*-test (n = 6–9).

**Table 2:** The EC_50_ and E_max_ of *B. monnieri* compounds on relaxations of endothelial intact (+EC), denuded (-EC) mesenteric arterial rings or endothelial intact arteries with L-NAME.

Active Compounds	EC_50_ (µM)	Emax (%)	n
**Luteolin**			
+EC	4.35 ± 1.31	99.35 ± 0.66	6
-EC	21.90 ± 5.86 †	82.42 ± 4.65 ††	6
+EC plus L-NAME	14.99 ± 3.56 †	90.85 ± 5.85	6
**Apigenin**			
+EC	8.93 ± 3.33	95.27 ± 2.61	9
-EC	12.80 ± 2.54	98.81 ± 1.19	8
+EC plus L-NAME	25.62 ± 3.38 ††	94.40 ± 2.10	7
**Bacoside A**			
+EC	10.81 ± 5.95	83.60 ± 2.86	7
-EC	14.50 ± 6.30	37.90 ± 4.72 ††	6
+EC plus L-NAME	33.81 ± 6.25 †	33.16 ± 8.41 ††	5
**Bacopaside I**			
+EC	14.63 ± 5.36	79.94 ± 8.17	7
-EC	17.29 ± 4.75	58.97 ± 7.05 †	7
+EC plus L-NAME	25.38 ± 4.33	58.45 ± 4.21 †	7

Comparison of EC_50_ or E_max_ of each component +EC vs. -EC or +EC plus L-NAME. † *p* < 0.05, †† *p* < 0.01 using unpaired Student’s *t*-test.

## Supplementary Materials

Supplementary materials are available online. Figure S1: Representative HPLC-UV chromatogram of mixed seven standards at 20 µg/mL for 1 and 2 and 100 µg/mL for 3–7 (A) and Brahmi extract (2 mg/mL) (B); luteolin (1), apigenin (2), bacoside A3 (3), bacopaside II (4), bacopaside X (5), bacopasaponin C (6) and bacopaside I (7), Table S1: Amount of each compound in 95% ethanolic extract of Brahmi analyzed by HPLC.
