# Supplementary material for: Vasodilatory Effects and Mechanisms of Action of Bacopa monnieri Active Compounds on Rat Mesenteric Arteries
Source: Molecules. 2019 Jun 15;24(12):2243. doi: 10.3390/molecules24122243 (PMC6630913; doi:10.3390/molecules24122243)
Supplement: Supplementary file 1 [file molecules-24-02243-s001.pdf]

Supplementary documents

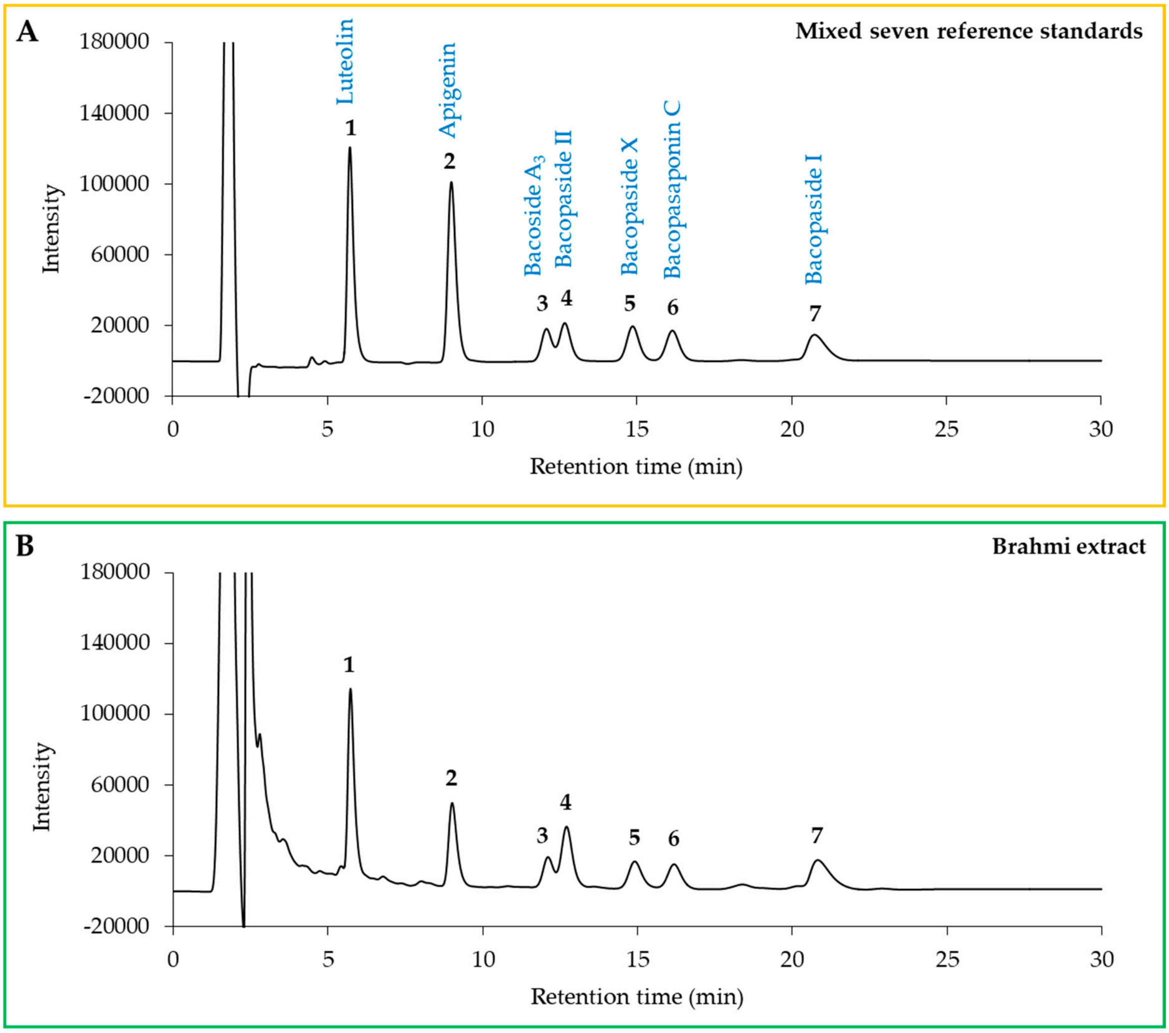

Figure S1. Representative HPLC-UV chromatogram of mixed seven standards at 20 μg/ml for 1 and 2 and 100 μg/ml for 3–7 (A) and Brahmi extract (2 mg/ml) (B); luteolin (1), apigenin (2), bacoside A3 (3), bacopaside II (4), bacopaside X (5), bacopasaponin C (6) and bacopaside I (7). The HPLC method followed the previous report [1].

Table S1. Amount of each compound in 95% ethanolic extract of Brahmi analyzed by HPLC. The values are expressed as averages from triplicate experiments ± standard deviations.

**Compound**	**Amount** (mg/g of dried extract**)**
Luteolin	1.39 ± 0.07
Apigenin	0.77 ± 0.06
Bacoside A_3	9.16 ± 0.13
Bacopaside II	15.63 ± 0.53
Bacopaside X	7.07 ± 0.36
Bacopasaponin C	8.19 ± 0.38
Bacopaside I	10.69 ± 0.19

Total flavonoids ~2.1 mg/g; total saponins ~51 mg/g

References

1. Saesong, T.; Temkitthawon, P.; Nangngam, P.; Ingkaninan, K., Pharmacognostic and physico-chemical investigations of the aerial part of *Bacopa monnieri* (L.) Wettst. *SJST* **2019**, 41, 397-404.
